# Medication assisted treatment (MAT) in criminal justice settings as a double-edged sword: balancing novel addiction treatments and voluntary participation

**DOI:** 10.1186/s40352-020-0106-9

**Published:** 2020-03-14

**Authors:** Jordan M. Hyatt, Philipp P. Lobmaier

**Affiliations:** 1 Department of Criminology and Justice Studies, Drexel University, 3141 Chestnut Street, Philadelphia, PA 19104 USA; 2 Norwegian Centre for Addiction Research, University of Oslo, Kirkeveien 166, building 49, Oslo, 0450 Norway

**Keywords:** Medication-assisted treatment (MAT), Opioid abuse, Corrections, Drug addiction, Informed consent, Coercion

## Abstract

Medication-Assisted Treatment (MAT) provides an opportunity to address opioid addiction among justice-involved individuals, an often difficult to reach population. This potential has been increasingly recognized by agencies, policymakers and pharmaceutical companies. The result has been a marked increase in the number of drug courts, prisons and agencies in which MAT, notably with long-acting injectable medications, is offered. While this is a positive development, ensuring that vulnerable individuals are in a position voluntarily participation within the complex criminal justice environment is necessary. The unequal authority and agency inherent in the nature of these environments should be recognized. Therefore, rigorous protections, mirroring the goals of the consent processes required for medical or sociobehavorial research, should be employed when MAT is offered to protect individual autonomy.

## Introduction

The number of individuals dying from opioid or opioid-related overdoses represents an ongoing societal challenge. In December 2017, for example, recent reports from the American Centers for Disease Control and Prevention (CDC) indicated that 47,855 individuals were reported to have died from opioid-involved overdose in the 12 months prior (Ahmad, Rossen, Spencer, Warner, & Sutton, 2019). Among those deaths, 28,659 individuals were identified as having overdosed on synthetic opioids and 15,593 were attributed to heroin (Ahmad et al., 2019). Over the previous year, this represents a 6.6% increase for the United States overall, with some states reporting increases as high as 33.3% (Ahmad et al., 2019). Unsurprisingly, opioid abuse has been classified as an urgent public health crisis (Kolodny et al., 2015). These challenges are not limited to the United States (e.g., Norway: Ødegård, Amundsen, & Kielland, 2007), though the scale and rapid increase in the scope of the public health crisis may be unique.

The impacts of opioid use disorder are also reflected in the composition of correctional populations, both in terms of the number of inmates with a history of use and in the number of sentences for drug-related offenses (Bukten et al., 2011; Mumola & Karberg, 2006). Despite this, traditionally, criminal justice agencies have not taken an active role in treating or preventing opioid use (Brinkley-Rubinstein et al., 2018), notwithstanding high rates of addiction observed among inmates (Compton, Dawson, Duffy, & Grant, 2010; Mumola & Karberg, 2006). This responsibility was seen as the purview of public health and community-based treatment providers. Recently this balance has shifted in part due to the ongoing opioid crisis which has brought about increasingly serious addiction issues in the community and has contributed to growing correctional populations generally, especially in the number of inmates with a history of opioid use.

Correctional agencies in general, and drug courts in particular, have been increasingly focused on the development of addiction-centric supervision and specific sentencing options for people who use drugs (PWUD). Novel MAT medications, such as long-acting Vivitrol, have been added to these programs. The information available is derived from a small number of academic evaluations and the potential for undue influences to impact defendant’s decisions to participate have not been fully explored. In this new context, therefore, effective responses to the epidemic should also address and properly evaluate the unique challenges, including voluntary consent, inherent in justice system-based addiction treatment interventions.

## Background

Despite recent, drastic shifts in pattern of drug use and overdose, the challenges posed by opioid abuse are not new and a multitude of behavioural and pharmacological treatments have been developed since the 1970s (Connery, 2015; Dugosh et al., 2016; Timko, Schultz, Cucciare, Vittorio, & Garrison-Diehn, 2016). In the past few years, however, several new options for medication-assisted treatment (MAT) have emerged, one of the most prominent options being the FDA approved extended-release naltrexone (Vivitrol), a once-monthly injection. MAT provides a pharmacological treatment to address the physiological aspects of addiction and withdrawal and is often delivered in conjunction with behavioural therapies. In addition to the immediate physiological benefits, MAT with methadone or buprenorphine maintenance has been shown to effectively stabilize addicted lifestyles in the longer term with reductions in crime rates, infections and overdose mortality (Ayanga, Shorter, & Kosten, 2016; Connery, 2015; Lobmaier, Gossop, Waal, & Bramness, 2010). MAT prevents relapse to daily heroin use, keeps PWUDs in treatment and is often the recommended first choice treatment for PWUDs in the community (Dugosh et al., 2016, Hedrich et al., 2012, Timko et al., 2016, World Health Organization, 2009).

However, less attention has been focused on evaluating and understanding the effects of these interventions specifically for justice-involved populations. The availability of limited evidence is of concern when introducing a novel treatment for a specific population that may respond differently or in a new context; the additional of long-acting MAT into drug courts provides such an example. Although there is a growing body of research focusing on MAT in drug courts (Matusow et al., 2013), and although criminal justice outcomes are increasingly acknowledged as important outcome measures (e.g., Lee et al., 2016), the rate of new studies within the literature with this specific focus have not kept pace with the expansion of novel MAT interventions within various phases of the criminal justice process.

Addiction poses a distinct challenge for justice-involved individuals; common correlates of addiction, including poverty, lack of access to healthcare and mistrust of the medical system are often overrepresented in this population. Access to effective substance use disorder treatment while in contact with the CJ system varies meaningfully between jurisdictions and is generally less prevalent than is available to PWUDs residing in the community. This differential contributes to the higher rates of both attrition from drug treatment and eventual relapse observed in justice-involved individuals (Wooditch et al., 2018).

Though the mechanisms are complex, there is a clear and persistent relationship between opioid use and criminal activity. Several studies have shown that there is an increased risk of death due to drug overdose during re-entry, especially in the time period immediately after an individual is released from incarceration (e.g., Merrall et al., 2010). While working within the criminal justice system creates an opportunity to deliver potentially lifesaving treatments to a population that can be difficult to access reliably, PWUD are often underserved in the criminal justice system and an increase in the number novel responses needs to be accompanied by proper, voluntary evaluation of its effects. Introducing new treatments or increasing access to established treatments in a new setting presents an increasingly complex ethical dilemma by justice agencies actively engaged in providing or facilitating access to MAT for opioid addiction (See Chandler, Fletcher, and Volkow (2009) for an overview of the addiction-related findings related to drug disorders treatment for justice-involved populations).

## Medication-assisted treatment options for opioid addiction

The common goal of treating opioid dependence with medications of any kind is to substitute the short-acting, high-potency opioid (which is often injected) with a non-illicit replacement. Some MAT options can block the user from feeling the effects of additional opioids, while others limit the physiological and physical symptoms of withdrawal. Behavioural therapy, either in the form of counselling or group therapy, is often contemporaneously engaged in. There are multiple medications that can be used as part of a MAT program, including heroin (which is uncommon), methadone and buprenorphine (among the most common globally), though each works differently (Krupitsky, Blokhina, Zvartau, & Woody, 2017). For example, some medications are agonists in that they fully or partially activate the same receptors in users’ brains as opioids. Others (such as naltrexone) are antagonists, acting to block any other chemicals from activating those same opioid-specific receptors. Table 1, below, details several commonly employed MAT medications, many (though not all) have been used in a criminal justice setting across the globe.

**Table 1:** Medication-assisted treatment (MAT) for opioid addiction: primary options

Active ingredient (Brand name)	Available as injection?	Addictive potential	Dosing scheme	Reversible?	Available in the USA / EU?
Methadone	No	Yes (agonist^a^)	Once daily	Yes, subject to 5–10 days of withdrawal	Yes / Yes
Buprenorphine (Subutex, Suboxone, Buvidal)	Yes	Yes (partial agonist / antagonist^a^)	Daily or thrice weekly^b^	Yes, subject to 3–8 days of withdrawal	Yes / Yes
Naltrexone (Vivitrol)	Yes^c^	No (antagonist^a^)	Every 4 weeks	No	Yes / No, but ongoing studies

Currently, maintenance treatment with Methadone or Buprenorphine is generally the first choice of treatment for opioid addiction. There is less supporting evidence for maintenance with injectable diacetylmorphine, long-acting morphine tablets or oral naltrexone. However, injectable naltrexone appears superior to tablets and is comparably effective to Buprenorphine-naloxone

^a^Mechanism of action at opioid receptors

^b^Injectable buprenorphine is given once weekly or once monthly

^c^Long-acting implants (Prodetoxone) are registered in the Russian Federation, whereas methadone and buprenorphine are prohibited

All agonistic medications have the potential for abuse as they serve to activate the opioid receptors. This means they likely have a street value and thus run the risk of illegal diversion (Johnson & Richert, 2015; Larance et al., 2014; Winstock & Lea, 2010). Therefore, MAT agonist dosing needs to be monitored and requires medically-trained oversight. Some MAT medications require frequent, in some cases daily, dosing, while others must be injected. Both pose unique challenges in justice settings, especially custodial environments.

An altogether different approach to MAT lies in substituting an agonist with an antagonist, receptor-blocking agent such as naltrexone (Kunoe, Lobmaier, Ngo, & Hulse, 2014). When provided in an extended-release preparation (currently available predominantly in the U.S. under the brand name Vivitrol), injectable naltrexone is a promising, if potentially underutilized, treatment for opioid addiction (Jarvis et al., 2018; Krupitsky et al., 2017; Volavka, Resnick, Kestenbaum, & Freedman, 1976).

The pressures of the present opioid crisis have accelerated the adoption of naltrexone within the general addiction and recovery communities, though this particular treatment option represents only a fraction of all cases in which addiction treatment is required (Chandler et al., 2009). Long-acting naltrexone formulations are promising options in the community (see e.g., Lobmaier, Kunøe, Gossop, & Waal, 2011). One systematic review identified positive effects for extended-release injectable naltrexone. After reviewing studies conducted between 2006 and 2017, the authors concluded that, although there are still few randomized evaluations comparing injectable naltrexone to either placebo or the current standard of care (6 of 22 eligible studies), there is evidence supportive of the clinical efficacy of the treatment, though challenges with adherence to treatment programs and variability in the detoxification status of participants within included studies warrants further examination (Jarvis et al., 2018).

Extended-release injectable naltrexone has several characteristics that make it appealing for justice-involved populations. In particular, injectable naltrexone is administered by a medical professional on a monthly basis and so cannot be diverted for resale (Festinger, Dugosh, Gastfriend, & Sierka, 2017). This sets this MAT apart from many other options, including methadone, which require daily dosing and can be (and often are) misused (see Table 1, above). This avoids the potential for abuse, but it also means that, once treatment has begun, it cannot be ceased immediately, an option for daily-dosed MAT medications.

Preliminary evidence from criminal justice settings supports the assertion that injectable naltrexone is viable MAT for reducing substance abuse (Coviello et al., 2012), costs (Murphy et al., 2017) and, in some cases, self-reported recidivism. Lee et al. (2016), for example, found that among volunteers with a criminal history after 24 months, 43% of the naltrexone group experienced a relapse compared to 64% of the control group (*p* < 0.001). Longer follow-up periods, however, found no significant differences in relapse nor were significant differences in incarceration rates identified.^1^ Based partially on these limited findings and partially on increasing public demand, a number of drug courts have begun to offer (and may sometimes formally or informally require) (Farabee, Prendergast, & Anglin, 1998; Woods & Joseph, 2015) treatment with extended-release injectable naltrexone (Belenko, Hiller, & Hamilton, 2015; Matusow et al., 2013). According to one relatively recent survey, 18% of drug courts offered some form of MAT treatment with naltrexone (with injections representing only 8 %), while well-established agonists were offered in 56% of these courts (Matusow et al., 2013). The “market share” of extended-release injectable naltrexone has almost certainly increased over the interim years, exposing an increasing number of defendants to the treatment option. In some instances, well-established MAT or behavioural therapy programs may be replaced by these newer programs, potentially limiting choice and flexibility, and without sufficient evidence of superior effects.

In addition to drug courts, MAT has been accepted by a wide range of other criminal justice agencies, with injectable or long-acting treatments recently supplementing the commonly used drugs (see Table 1). The adoption of these medications, especially market-leader Vivitrol, has been facilitated through public relations and marketing campaigns targeting justice agency officials with the specific goal of increasing the usage by other specialized courts (e.g., those for veterans or general re-entry), prisons and other agencies (Goodnough & Zernike, 2017; MacGillis, 2017). There are marketing materials specifically targeting criminal justice professionals (Harper, 2017; Walsh, 2017) and which tout the number of states, re-entry programs and specialized court programs in which forms of MAT, including injectable naltrexone formulations, have already been offered.

Given the wide range of court types in which MAT generally, and extended-release injectable naltrexone specifically, could be used, it is unsurprising that there is a high degree of variation in how treatment programs are managed, which participants are enrolled in them, and the consequences of treatment (or, more importantly, failure to comply with treatment) for supervision. A 2017 study by Physicians for Human Rights (PHR) is illustrative of these concerns. After examining the practices in drug courts in three states, they found significant variation in how medical decisions were made, including determinations by non-clinical staff, in levels of staff knowledge and acceptance of MAT and in how treatment options were communicated. Additionally, these jurisdictions had different policies for how treatment was to be paid for, participant obligations and how violations were to be addressed. These barriers are facilitated, as the PHR report notes, by the tensions between correctional ideologies focused on following rules and the therapeutic approach that characterizes addiction treatment in most other contexts (Physicians for Human Rights, 2017). While this report should not be taken to suggest that all criminal justice-based programs that employ extended-release injectable naltrexone operate in a problematic manner, it does make apparent several potential complications. It is clear from PHR’s conclusions that courts are not medical centres; judges and administrators are not doctors. While there are many shared goals, there may also be tensions between these two frameworks, with vulnerable individuals potentially caught in the middle (Chandler et al., 2009).

An examination of the literature on extended-release injectable naltrexone, including recent Vivitrol-focused trials, within the criminal justice system, as discussed above, is illuminating. All of the relevant, peer-reviewed studies on justice-involved populations have been conducted by academic researchers in collaboration with prisons, courts or other agencies. There are, however, hundreds of drug court and other programs where extended-release injectable naltrexone is provided or required. While biases in research and publication may underrepresent the scope of robust community-initiated programming, and there may be stark differences between externally evaluated and internally developed MAT programs. We highlight one such potential challenge here.

## A particular challenge for criminal justice systems: the potential limits of voluntariness in participation

The *Belmont Report*, which provides influential guidelines for clinical research, sets out the need to ensure voluntary participation as a key element of ethical research. In its summary, the Office for Human Research Protections at the U.S. Department of Health and Human Services (1979), defines these challenges as follows:

*Coercion occurs when an overt threat of harm is intentionally presented by one person to another in order to obtain compliance. Undue influence, by contrast, occurs through an offer of an excessive, unwarranted, inappropriate or improper reward or other overture in order to obtain compliance. Also, inducements that would ordinarily be acceptable may become undue influences if the subject is especially vulnerable … Unjustifiable pressures usually occur when persons in positions of authority or commanding influence -- especially where possible sanctions are involved -- urge a course of action for a subject.*

Wertheimer notes that coercion can undermine the legally transformative value of consent (Wertheimer, 1996), effectively undermining the ethicality of the otherwise non-exploitative treatment. While coercion can be limited to overt threats, Wertheimer also argues that this level of influence is also present when an individual is presented with a set of options, within a forced choice context, where there is no reasonable option but to submit to treatment (or whatever the will of the coercer might be). The lack of consent, either when not obtained or when invalidated through the impact of coercion (or the softer pressures of undue influences), makes a proposal exploitive, even when the subject objectively benefits (Wertheimer, 1987, pp. 222–241).

Within an addiction treatment context, coercion and undue influence present a complex paradigm. It is clear that treatment benefits the recipient. However, even participants who voluntarily begin drug treatment can report feeling coerced into participating (Damon et al., 2017). A study comparing voluntary participants in drug treatment to legally, but involuntarily, enrolled participants in Norway found perceptions of coercion in both groups, though the involuntary group attributed this influence to the legal system as compared to internal influences (Opsal, Kristensen, Vederhus, & Clausen, 2016). From an empirical perspective, the extent of legal and informal coercion can have an impact on the efficacy of drug treatment; the results are sometimes negative (Wegman et al., 2017), though not exclusively (Anglin, Brecht, & Maddahian, 1990). A study by Coviello and colleagues found that when offenders were mandated to participate in a intensive cognitive-based therapy program, they were demonstrably less motivated, but, these court-ordered participants were ten times more likely to remain in treatment (OR = 10.9, *p* = .006) (Coviello et al., 2013). In other cases, individuals undergoing mandatory treatment have performed better than comparable volunteers in treatment (Kelly, Finney, & Moos, 2005). At the same time, individuals who have been coerced into treatment, even by legally permissible means, may still perceive their participation as voluntary (Young, 2002; Young & Belenko, 2002). This does not mean that coercion and undue influence are inherent and preclude treatment, only that they should be considered as potentially meaningful part of the context (Wolfe, Kay-Lambkin, Bowman, & Childs, 2013).

The actively justice-involved population should be considered distinctly with regard to the pressures to enroll in treatment. They are, after all, distinguishable in many ways from an average group of individuals who have similar medical diagnoses or addiction histories. The well-established correlates of incarceration include non-representative experiences with poverty, systematic disadvantage and education, all of which may impact a given individual’s ability to appropriately comprehend the basic requirements of consent. Higher levels of certain conditions, including mental illness, traumatic brain injury and, of course, addiction, also negatively influence cognitive processes and may challenge the ability of some justice-involved individuals to fully engage in the court process. Additionally, the pressures of a courtroom or prison environment and the legal penalties for non-compliance (or even the appearance of such), set a different set of risks and benefits.

A drug court program, or any other criminal justice-focused treatment program is not, in itself, inherently coercive, though they are designed to encourage treatment (Burns & Peyrot, 2003). As package of supervision and treatment paradigms, these have become some of the most effective relapse- and recidivism-reduction strategies available today (Jewell, Rose, Bush, & Bartz, 2017; Lowenkamp, Holsinger, & Latessa, 2005; Mitchell, Wilson, Eggers, & MacKenzie, 2012). Additionally, participants often see these programs as more procedurally just and fairer than the alternatives (Gottfredson, Kearley, Najaka, & Rocha, 2007).

Issues relating to coercion and treatment are not unique to the criminal justice context nor to any particular jurisdiction or program. Debates about involuntary hospitalization for some forms of mental health treatment have been ongoing (Monahan et al., 1995), though this remains a legal option in many cases of severe illness, distress or danger (Simon & Rosenbaum, 2015). Using civil mechanisms, for example, involuntary commitment to inpatient treatment is an option of last resort for some conditions, including eating disorders (Douzenis & Michopoulos, 2015; Yager, Carney, & Touyz, 2016).

The relationship between individual autonomy and mandatory long-acting naltrexone treatment is complicated. Issues of autonomy, agency and the nature of sobriety are implicated (Caplan, 2006). While talk-based therapy has long been mandated (for example, anger management or parenting classes as a condition of probationary supervision), the pharmacological nature and potential side effects of MAT provide a distinctly different landscape. Extended-release naltrexone, once initiated, precludes withdrawal from treatment during the first month while the injection exerts its effect. This requires explicit information through an informed consent procedure. To date, there appears to be significant variation in how drug courts offering Vivitrol as an option during sentencing or supervision inform participants about the irrevocable nature of Vivitrol and the philosophical and medical debate around these issues. The potential vulnerability of justice-involved populations makes this matter particularly pressing and warrants involvement of individuals who have experience with such communication strategies, including informed consent procedures.

It is difficult to overstate the extent to which, within the criminal justice context, the balance of power is significantly more unequal than in general treatment settings (Marlowe, 2006). Judges in drug courts, as well as probation officers supervising individuals in recovery while under community supervision, wield an enormous amount of authority. In particular, each correctional party has the ability, with little formal oversight, to potentially send a defendant or probationer to prison for failing to comply with treatment or for using illegal drugs, the focus of their disease. Defendants and parolees, on the other hand, lack agency in this process and, in the case of community-based programs, may have recently experienced the effects of opioid abuse, withdrawal or the influence of other physical and psychological effects of drug use.

The courtroom environment is complex, leaving many defendants unclear as to their rights and obligations. Drug courts, with their focus on non-mandatory enrolment in programming are, in some cases, explicitly designed to be coercive (Burns & Peyrot, 2003). Even when they are not, criminal justice populations with an addiction history, due to unique but shared drug, medical and psychosocial characteristics, may not be well equipped to understand their ability to deny consent to treatment in this environment (Ahalt et al., 2017). While this may be acceptable for well-proven, behavorial or cognitive skills-based interventions that do not directly influence brain physiology, it is problematic when there is a need to ensure that receipt of long-acting pharmacological treatment is truly both knowing and willing.

Guidelines for clinicians (e.g., Kampman & Jarvis, 2015) and drug court officials (e.g., Jushner, Peters, & Cooper, 2014) often acknowledge the general need for obtaining consent prior to MAT treatment and the general need for voluntariness. The recommendations undoubtedly follow best-practices with regard to clinical treatment. What is lacking, however, is an overt recognition that the environment within a criminal justice agency is different than that of a hospital or clinic, especially when a potentially life-saving treatment is being offered in an environment as complicated as a courtroom or a jail and where the penalties for noncompliance may include being sent back to prison.

## A framework for a potential solution

Informed consent and voluntariness are of concern in medical and socio-behavioural research with human subjects (Flory & Emanuel, 2004). These complex processes are implemented, and are mandatory in most cases, to protect vulnerable participants from being influenced, potentially against their will or best-interests, to participate in research studies. Though several decades in the past, the influence of the Tuskegee and Holmsberg Prison experiments, among others studies conducted on a coerced or uninformed prison population (Green, Maisiak, Wang, Britt, & Ebeling, 1997; Hornblum, 1997; Washington, 2006), can be seen reflected within the requirements for research with justice-involved and other vulnerable populations (e.g., U.S. Department of Health and Human Services, 1979).

MAT-focused programs, especially those using long-acting medications like injectable naltrexone, must take into account that drug courts, and the criminal justice system generally, present a complicated environment for addiction treatment delivery. In this sensitive setting, relapse or failure to comply with treatment may subject defendants to additional punishment. This may constitute a form of undue influence in some cases.

The growth of programs using Vivitrol, and the publicity accorded these programs due to the manufacturer’s marketing efforts and media attention driven by the underlying public health crisis, provide an example of where such challenges may flourish. While a small percentage of pilot programs have been independently and rigorously evaluated as part of externally-funded research studies, (e.g. Lee, 2016), many new Vivitrol-based programs for defendants are not (Finigan et al., 2011; Griffith, 2017; MacGillis, 2017; Remoquillo & Ohio, 2015). This suggests that the level of transparency and accountability required for research purposes may be lacking in a significant number of the day-to-day administrations of Vivitrol within the criminal justice system. While the system is invested in the treatment option, the knowledge of participants and their voluntariness under pressure (even well-intentioned) is unclear. This opaqueness undermines confidence in the ethicality and fairness of these programs, despite their laudable goals.

Coviello et al. (2012) illustrate the importance of following best-practices in their description of efforts to recruit subjects for a study on another preparation of extended-release injectable naltrexone. In that instance, they sought to recruit a convenience sample of probationers and parolees. They note that, when speaking with potential participants:

*Special efforts were made to assure offenders that participation in the research study was voluntary, and they were instructed that participation in the research was an additional service they could receive. All potential subjects were informed that choosing to participate or not participate in the trial would have no effect on their probation or parole status and they could stop participation in the study at any time without affecting their treatment or criminal justice status.*

While this scope of consent is the norm for research studies governed and monitored by IRB and federal regulators, a court-based program is not necessarily bound by these same rules. While the IRB system is imperfect, it provides a foundational framework onto which progress can be built. An outside oversight agency, like an IRB, provides for an opportunity to assess threats to voluntary participation, review relevant procedures and ensure compliance, all to protect participant autonomy (see e.g., Largent, Grady, Miller, & Wertheimer, 2013). While many programs can, and probably do, follow these types of procedures, adherence to general principles encourages uniformity in treatment and opportunity, as well as protects participant autonomy. A reliance on this conceptual and practical framework supports several broad principles important for both the both correctional oversight and addiction treatment:

### Increase the opportunity to provide MAT

Opioid-addicted defendants, inmates and parolees should generally be entitled drug treatment options that are similar to those offered outside of the criminal justice context (Bruce & Schleifer, 2008), though the unique nature of the justice-system will often influence which MAT options are given and when they are made available. This challenge should not allow for the depravation of treatment for those in need; established MAT programs should be allowed, and often encouraged, to flourish. In light of the opioid crisis, criminal justice and judicial entities should continue to facilitate treatment with the most effective options available to them.

### Acknowledge the potential for coercion

There is an imbalance of power inherent in the relationship between criminal defendants and criminal justice system authorities. This disparity increases the risk for coercion and the processes that parallel researcher-led studies with informed consent procedures can protect subjects from coercive pressures. While these procedures are imperfect, they provide a framework and direction for progress. This addition, again, should not preclude MAT treatment nor distinguish how justice-involved populations are treated from those voluntarily seeking drug treatment in the community.

### Obtain a form of informed consent

Mirroring the procedures required for research, defendants, inmates and parolees who are eligible for MAT treatment should be required to formally and officially indicate their willingness to participate prior to initiation of treatment. There are myriad ways to accomplish this in a manner that does not impede treatment delivery. At a minimum, this explicit and ongoing consent should be obtained from all participants to support the assumption that their involvement is voluntary and not the result of an environment that is intentionally or unintentionally coercive. Informed consent implies the possibility to withdraw from study treatment at any time without stating reasons and without consequences, especially in regards to incarceration or the terms of their sentence. This will require a robust effort to educate the potential participants about their rights and autonomy, as well as the potential risks and benefits of MAT treatment within that specific criminal justice context. These processes should account for the common challenges (e.g.*,* literacy level) found within these populations (Cislo & Trestman, 2013; Edens, Epstein, Stiles, & Poythress Jr, 2011).

### Encourage independent oversight

While an oversight role can be occupied by an Institutional Review Board (IRB) or other administrative body run by an academic institution, research centre or third party, correctional agencies can develop similar boards. Many such review committees are already in place and can serve as a model. Staffing these oversight entities with independent auditors and subject matter experts will increase the level of protection without unjustly hampering efforts to deliver potentially beneficial treatment.

### Staff programs for both justice and medical needs

Many correctional institutions and other long-term housing facilities, including in- and out-patient drug treatment programs, employ full time and certified medical staff. These doctors and nurses make individualized clinical decisions for MAT, oversee treatment, ensure compliance and seek to reduce attrition. While many drug courts have similar staff, these individuals should ensure that, when a MAT recommendation is made, it is in line with both acceptable clinical and criminal justice parameters as they continually evolve in light of new, relevant research.

### Develop the context-specific evidence-base

Providing potential MAT recipients with a complete understanding of the risk and benefit profile of treatment requires a robust understanding of the evidence base. Studies should address the unique context and populations that interact within criminal justice contexts. While there are an increasing number of studies relevant to MAT usage in this context, especially on programs offering Vivitrol, in the peer-reviewed literature, gaps remain. Encouraging collaboration between academic researchers with expertise in criminal justice and addiction-focused evaluations will accelerate this process, as well as bolstering the processes for securing informed consent more broadly.

These are significant undertakings, but they are not unsurmountable. Many correctional and judicial entities engaged in delivering, facilitating access to or mandating treatment have taken such steps already. There are many sets of guidelines that outline the policies that can, and should, govern the administration of drug courts. For example, Kushner, Peters, and Cooper (2014), provide a detailed set of procedures for the use of MAT in a drug court context. In the section entitled “‘Coerced treatment’ and the role of ‘motivation’”, they acknowledge the complex issues surrounding a willingness to participate in treatment. Expanding this concept to include concrete guidance as to what constitutes informed consent to receive a long-acting medication, what procedures could limit coercion, and how to transparently document this process, will only further the goals of these programs. The National Drug Court Institute (NDCI) standards address the usage of MAT and they have, in partnership with the American Society of Addiction Medicine, produced guides for counsellors, drug court staff and defendants (Stensland, 2017). NDCI Standards II discusses consent with regard to data sharing and randomized evaluations, but not in the context of participant education or enrolment. Another example, drafted by the Legal Action Center in New York and the Center for Court Innovation, provides examples from the state of New York and a set of generalizable best practices (Friedman & Wagner-Goldstein, 2017). Other actors within the criminal justice system, including members of the judiciary, the bar or correctional agencies, should seek out or develop guidelines to reflect the unique nature of the environments in which they mandate treatment and to protect participant autonomy.

## Conclusion

Treatment with pharmacotherapy within a criminal justice settings that is long-acting and temporarily irrevocable, such as extended-release naltrexone, must be secured through voluntary participation and verified using informed consent. Informed consent should be applied as a condition sine qua non, just as it would be in a research study conducted by an external investigator. Without informed consent that clarifies the multifaceted role of long-acting MAT in drug courts, offenders may be coerced, unintentionally or by well-intended design, into receiving long-acting medical treatment, without having an alternative choice and with a suboptimal level of setting-specific evidence that it is effective.

In a criminal justice context, an individual facing a recommendation or treatment may be less able to choose treatment freely; they should not be subjected to treatment mandated (nor offered) by a judge as a condition of supervision or an alternative to release under these circumstances. Therefore, a framework of protections that parallels those within research should be put in place when opioid-involved offenders are offered any novel treatment in a criminal justice setting. Implementing new pharmacological treatments with justice-involved populations inherently brings about novel challenges, along with the potential to deliver life-saving treatments to a difficult to reach population and during a challenging time. The additional costs required to ensure the protection of individual autonomy, choice and the avoidance of the appearance of coercion are well worth bearing.

## Data Availability

N/A

## Footnotes

1 The limited number of studies on justice-involved populations have been conducted in the community (Coviello et al., 2012), in drug courts (Finigan, Perkins, Zold-Kilbourn, Parks, & Stringer, 2011) and during an inmates return to the community after incarceration in prisons (Friedmann, Wilson, Hoskinson, Poshkus, & Clarke, 2018; Hyatt, 2017) and jails (Lincoln, Johnson, McCarthy, & Alexander, 2018). The wide range of contexts in which these studies have taken place reflect the potential depth of justice-system involvement in providing, or mandating, naltrexone-based MAT programs, as well as the need for context specific-replication and long-term follow-up of at least 6 months.
